# Laparoscopic approach in the management of diaphragmatic eventration in adults: gastrointestinal surgical perspective

**DOI:** 10.1007/s13304-023-01665-7

**Published:** 2023-10-17

**Authors:** El-Sayed Abou El-Magd, Ahmed Elgeidie, Amr abbas, Youssif Elmahdy, Ibrahem LotfyAbulazm, Hosam Hamed

**Affiliations:** Department of General Surgery, Faculty of Medicine, Gastrointestinal Surgical Center GISC, Mansoura University, Gehan Street, Mansoura, 35511 Al Dakahlia Egypt

**Keywords:** Diaphragmatic eventration, Laparoscopic approach, GIT symptoms, Laparoscopic plication, Gastric volvulus, Dual diaphragmatic pathology

## Abstract

The current literature is poor with studies handling the role of laparoscopy in managing diaphragmatic eventration (DE). Herein, we describe our experience regarding the role of laparoscopy in managing DE patients presenting mainly with gastrointestinal symptoms. We retrospectively reviewed the data of 20 patients who underwent laparoscopic diaphragmatic plication between January 2010 and December 2018. Postoperative outcomes and quality of life were assessed. Most DEs were left sided (95%). Laparoscopic diaphragmatic plication was possible in all patients, along with correcting all associated gastrointestinal and diaphragmatic problems. The former included gastric volvulus (60%), reflux esophagitis (25%), cholelithiasis (5%), and pyloric obstruction (5%), while the latter included diaphragmatic and hiatus hernia (10% and 15%, respectively).The average operative time was 142 min. All patients had a regular (reviewer #1) postoperative course except for one who developed hydro-pneumothorax. At a median follow-up of 48 months, midterm outcomes were satisfactory, with an improvement (reviewer #1) in gastrointestinal symptoms. Three patients (reviewer #1) developed radiological recurrence without significant clinical symptoms. Patient’s quality of life, including all parameters, significantly improved after the laparoscopic procedure compared to the preoperative values. Laparoscopic approach is safe and effective for managing adult diaphragmatic eventration (reviewer #1).

## Introduction

Diaphragmatic eventration is a rare pathological entity characterized by abnormal development of the diaphragmatic musculature leading to abnormal elevation of one or both hemidiaphragms. That entity could be congenital or acquired [1]. Although most cases are asymptomatic in early life, they could present later with respiratory or gastrointestinal manifestations [2–6].

The management of this problem varies according to its presentation. No intervention is required in asymptomatic cases. Nonetheless, diaphragmatic plication provides a good option for symptomatic ones [4].

The minimally invasive approaches are usually preferred for that intervention [3, 7]. The most commonly advocated one is thoracoscopic plication [3, 8]. However, the laparoscopic approach could have more benefits (reviewer #1). It offers better visualization and greater work space, avoids the potential risk of injury to unvisualized abdominal viscera by thoracoscopy, and can detect and repair the co-existing gastrointestinal (GIT) (reviewer #1) problems such as a gastric volvulus [3]. In addition, laparoscopy does not require the single-lung ventilation required during thoracoscopy [5].

Only small case series and case reports are available in the literature regarding laparoscopic management of diaphragmatic eventration in adults, especially when the patients present with GIT complaints [2, 9–11].

Herein, we present the benefits of laparoscopy in managing adult diaphragmatic eventration, and we will highlight its beneficial impact on the associated gastrointestinal symptoms.

## Patients and methods

Between January 2010 and December 2018, 20 adult patients diagnosed with diaphragmatic eventration underwent successful laparoscopic diaphragmatic plication at our Gastrointestinal Surgical Center, Mansoura University. After gaining approval from our local scientific committee (IRB code: R.22.09.1836), the data of these cases were collected from our database and then reviewed.

Our study included patients aged ≥ 18, diagnosed with diaphragmatic eventration, and presenting mainly with gastrointestinal symptoms.

Preoperative assessment included medical history taking, clinical examination, and routine laboratory investigations. Their preoperative quality of life was assessed via the Gastrointestinal Quality of Life Index [12]. A chest radiograph was ordered for all patients to show the side and the level of the eventration.

**Fig. 1:**
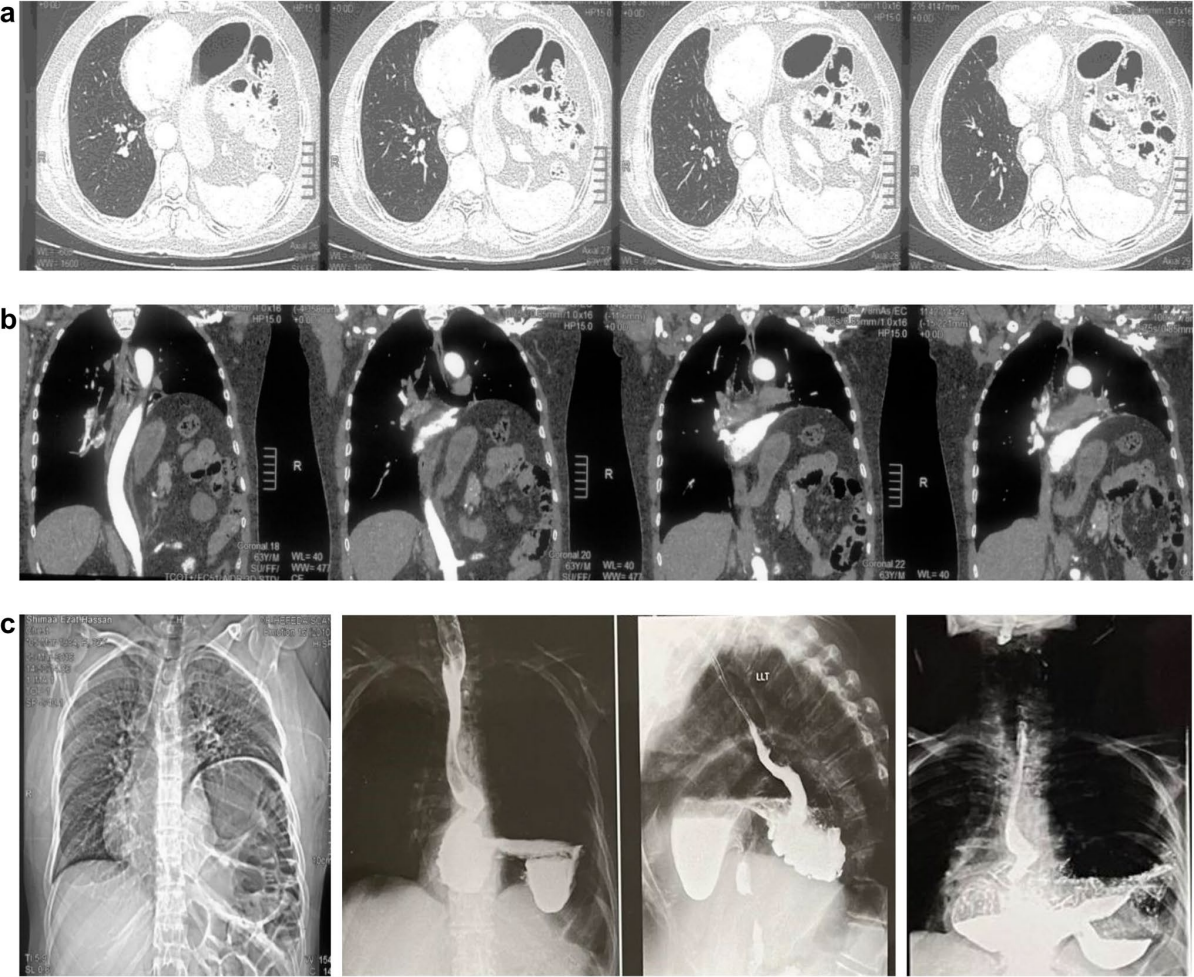
**A**, **B** CT evaluation of left diaphragmatic eventration. **C**, Chest X ray and barium study with finding of organaxial volvulus of the stomach

In addition, a barium meal, pelviabdominal ultrasound, and esophagogastroduodenoscopy were done to evaluate the reported gastrointestinal problems. Routine abdominal computed tomography (CT) was ordered in all cases to confirm the diagnosis (reviewer #1) (Fig. 1). If the patient reported associated pulmonary symptoms (e.g., dyspnea), pulmonary function tests were done. All patients were also reviewed by the anesthetic team and classified according to the ASA (American Society of Anesthesiologists).

All patients were managed via laparoscopy after signing an informed consent explaining that approach’s benefits and possible complications. Our laparoscopic management consisted of two main steps: the first was correcting the diaphragmatic problem, and the second was addressing the associated gastrointestinal problems.

## The surgical procedure

### Laparoscopic diaphragmatic plication

The laparoscopic procedure was performed when the patient was in a reverse Trendelenburg position with abducted arms. Abdominal insufflation was done via the Veress needle, followed by the entry of the periumbilical camera port. This was followed by the entry of two 5-mm working ports at the right and left midclavicular lines. Then, an epigastric port was inserted for liver retraction, and an assistant port was inserted in the left anterior axillary line.

**Fig. 2:**
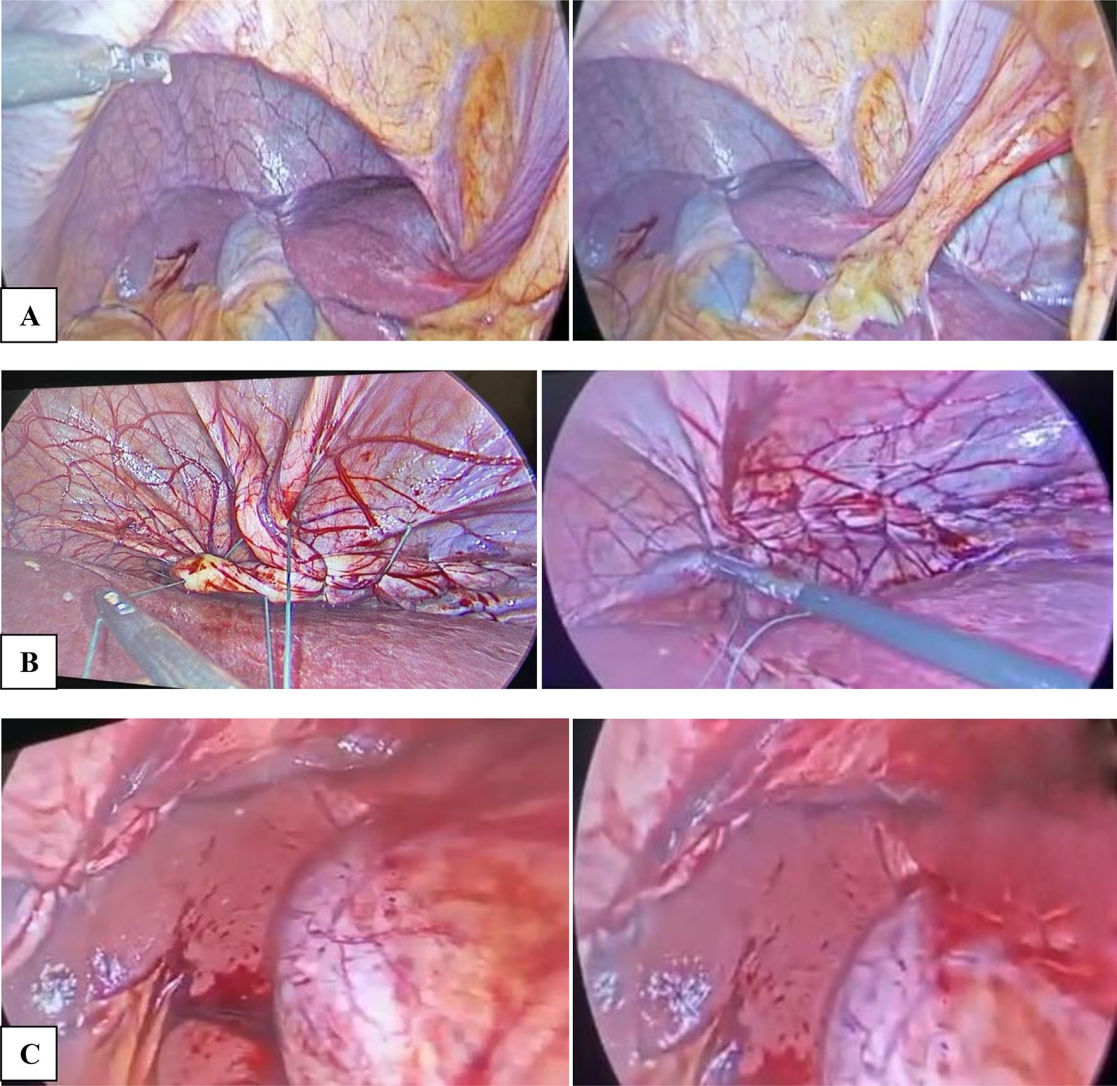
**A**, Right side diaphragmatic eventration. **B**, laparoscopic plication of diaphragmatic eventration. **C**, completed plication

**Fig. 3:**
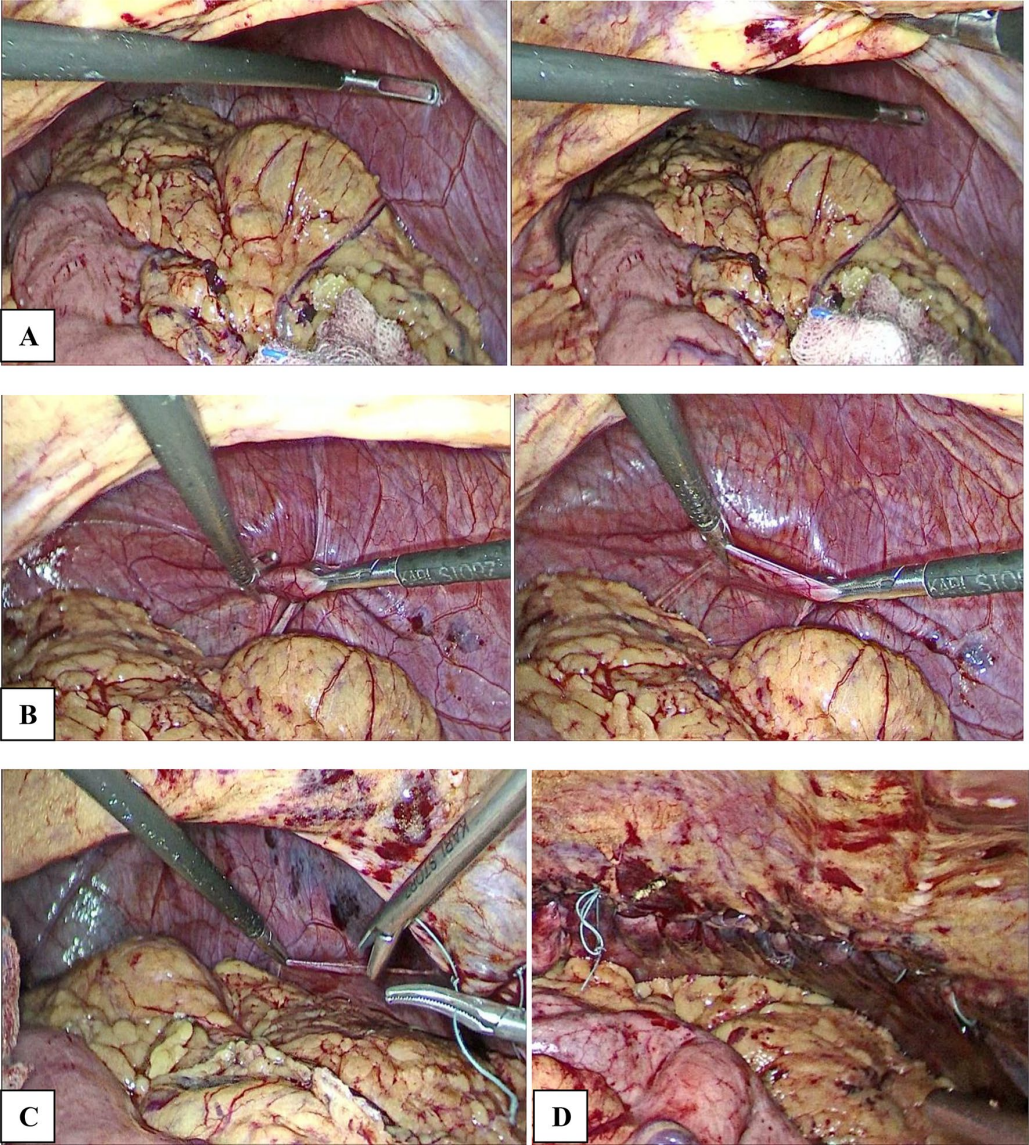
**A**, Left-sided diaphragmatic eventration. **B**, Catching the redundant eventration. **C**, Starting laparoscopic diaphragmatic plication. **D**, Completed plication of the eventration

The plication procedure was performed by folding the peripheral weak diaphragmatic area to the middle area, aiming to invaginate the diaphragmatic dome (Figs. 2 and 3). The plication was performed in layers with 2/0 polypropylene intracorporeal sutures. The plication was further supported with a layer of interrupted 2/0 Ethibond sutures. No mesh reinforcement was used. The target of the plication was to reduce the elevated loose diaphragm to a nearly normal level.

### Management of the associated GIT or diaphragmatic problems

#### GIT problems

**Fig. 4:**
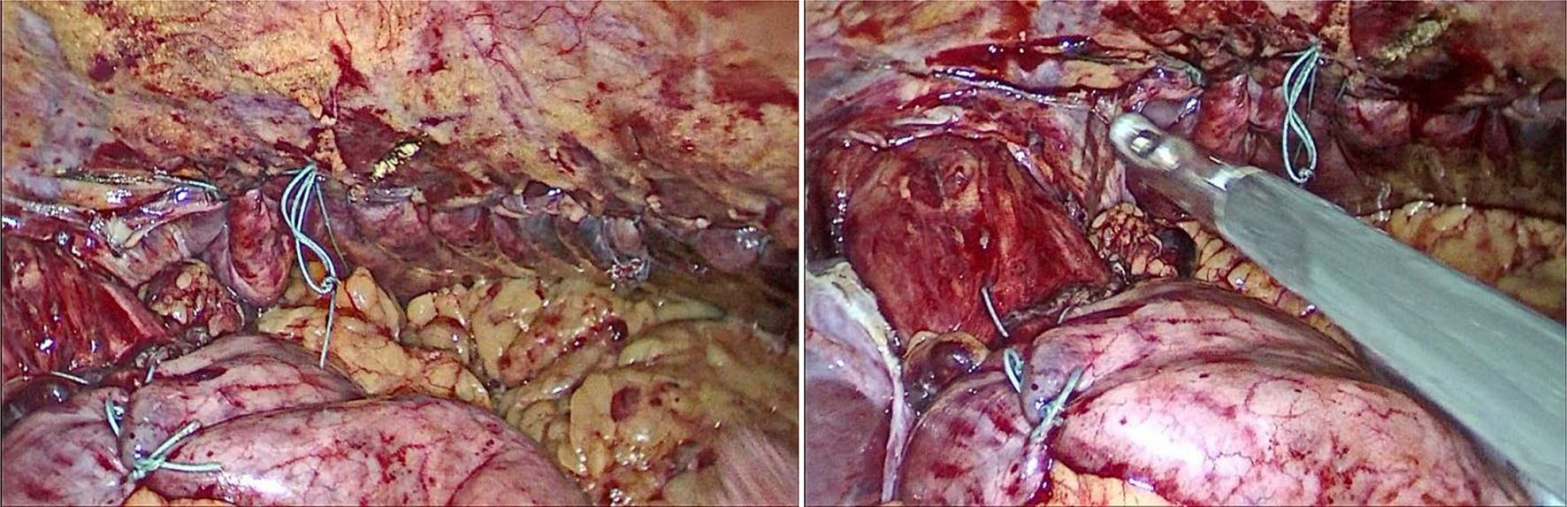
Plication and fundoplication

Some concomitant gastrointestinal problems were encountered in our patients, and they were managed during the same procedure. For chronic duodenal ulcers causing gastric outlet obstruction, a truncal vagotomy with simple loop gastrojejunostomy was done. If the patient had significant reflux manifestations, a crural repair with Nissen fundoplication was performed (Fig. 4). If a gastric volvulus was encountered, a posterior gastropexy was done by separating the stomach from the greater omentum and fixating its posterior wall to the nearby transverse mesocolon via interrupted non-absorbable sutures. We preferred this type of gastropexy over the anterior one (fixation to the abdominal wall) to avoid more tension and traction on the gastric wall (reviewer #2). In addition, laparoscopic cholecystectomy was performed if the patient had symptomatic cholelithiasis.

#### Dual diaphragmatic problems

**Fig. 5:**
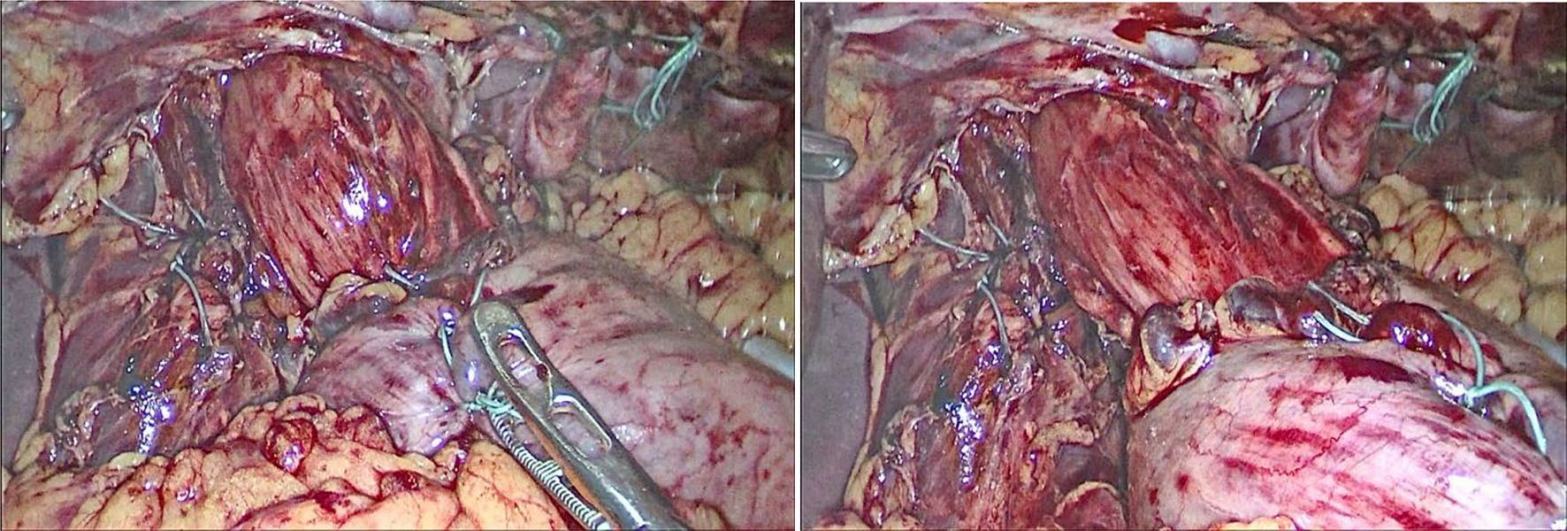
Repair of associated sliding hiatus hernia

Some patients also had other diaphragmatic pathologies (diaphragmatic and hiatus hernia) other than eventration. Diaphragmatic hernias were visualized at the center of the eventrated hemidiaphragm, and it was managed by the reduction of its contents and primary repair with interrupted non-absorbable sutures. If a sliding hernia was detected, it was managed by reduction of its contents, excision of the hernial sac, crural repair, and Nissen fundoplication [13] (Fig. 5).

### Postoperative care

All patients received the standard postoperative care, and oral fluid was usually allowed on the first postoperative day unless complications were encountered. Any postoperative complications were identified, recorded, and well-managed. After discharge, regular follow-up visits were scheduled to assess the improvement of patient symptoms.

### Follow-up

After discharge, the skin sutures were removed after 2 weeks, then the patients were followed at 1, 3, 6, and 12 months following the procedure. After that, yearly visits were scheduled. A chest radiograph was routinely ordered at the 3-month visit. In addition, a barium study or esophagogastroduodenoscopy was ordered yearly for investigational purposes or when the patient presented at follow-up with recurrent gastrointestinal problems. Postoperative quality of life was assessed after the operation, then after 1, 3, and 4 years using the same questionnaire applied preoperatively.

Recurrence was defined as clinical recurrence in the form of recurrent gastrointestinal symptoms, or radiological recurrence, defined as an elevation of the diaphragm of more than three intercostals spaces at follow-up chest radiographs.

Our main outcomes were quality of life changes and postoperative recurrence rates, whereas secondary ones included short-term postoperative complications.

### Statistical analysis

Categorical data were presented as numbers and frequencies, while continuous variables were expressed as mean (and standard deviation) or median (and range). Pre- and postoperative GIQLI scores were compared using the paired samples *t* test, while the one-way Anova test was used to compare the same score between three time points. The SPSS software (version 26 for macOS) was used to perform the previous tests, and any *p* value less than 0.05 was considered significant.

## Results

The basic demographic characteristics and the etiology of eventration in the included 20 patients are demonstrated in Table 1. We included 11 men and 9 women in our study. Their mean age was 50 years. Most of them had ASA class I (85%), and the remaining small ratio had ASA class II. Most patients had left-sided eventration (95%), while only one (5%) had a right-sided disease. Regarding the etiology of eventration, it was post-traumatic in 15 cases (75%), while the remaining 5 cases had idiopathic eventration.

**Table 1:** Demographic data and etiology of eventration in the study cases

Variable	Data (*n* = 20)
Age (years)	50 ± 11.5
Sex	
Male	11 (55%)
Female	9 (45%)
BMI (kg/m^2^)	26.3 ± 2.8
ASA class	
I	17 (85%)
II	3 (15%)
Affected side	
Left	19 (95%)
Right	1 (5%)
Etiology	
Post-traumatic	15 (75%)
Idiopathic	5 (25%)

The main presentation was upper gastrointestinal symptoms in the form of epigastric pain and vomiting that were present in most cases. Reflux symptoms were reported by five patients (25%). In only two patients, chronic dyspnea was associated with these symptoms (10%). At admission, one patient had persistent vomiting with epigastric fullness and was diagnosed with pyloric obstruction due to a chronic duodenal ulcer (Table 2).

**Table 2:** Clinical presentation of the study patients

Variable	Data (*n* = 20)
Chronic dyspnea and epigastric pain	2 (10%)
Epigastric pain and vomiting	12 (60%)
Reflux symptoms	5 (25%)
Persistent vomiting	1 (5%)

All patients were performed with laparoscopy with no need for conversion to the open approach. Multiple diaphragmatic and gastrointestinal pathologies were encountered during the laparoscopic assessment. Regarding the diaphragmatic ones, a diaphragmatic hernia was present in two patients (10%), while a hiatus hernia was present in three cases (15%). The latter three cases were among the five patients who presented with preoperative reflux symptoms. Regarding gastrointestinal problems, two patients had significant reflux manifestations; one had gallbladder stones (Table 3).

**Table 3:** Operative data

Variable	Data (*n* = 20)
Operative time (minutes)	140 ± 30.2
Intraoperative blood loss (ml)	130 (0–200)
Concomitant diaphragmatic lesions	
Diaphragmatic hernia	2 (10%)
Hiatus hernia with reflux	3 (15%)
Concomitant gastrointestinal pathologies	
Gastric volvulus	12 (60%)
Reflux with gallstones	1 (5%)
Reflux only	1 (5%)
Cicatrized duodenal ulcer	1 (5%)
Intraoperative complications	0 (0%)
Conversion to the open approach	0 (0%)

**Fig. 6:**
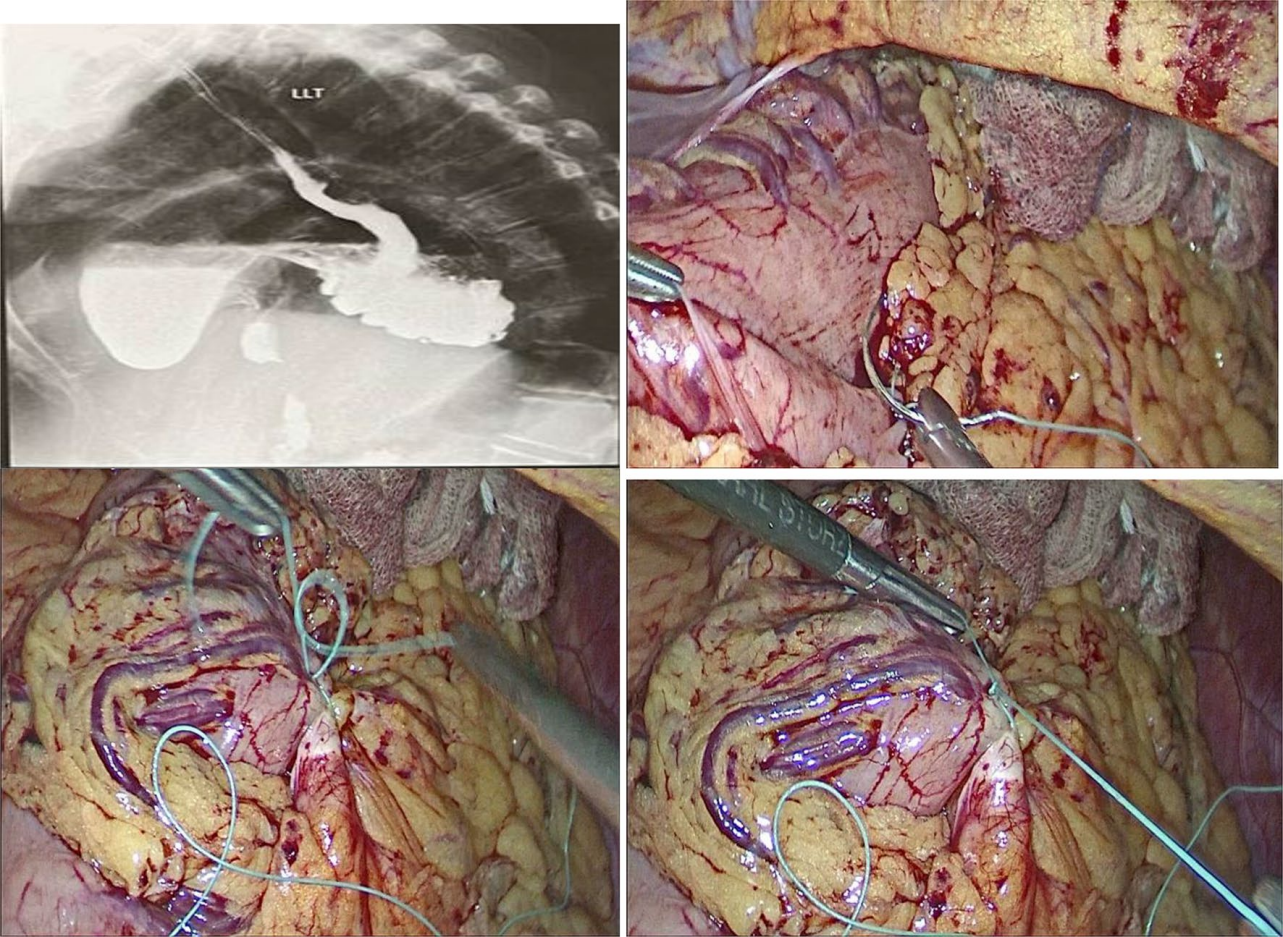
Posterior gastropexy for associated gastric volvulus

Pyloric obstruction was detected in one patient due to cicatrized duodenal ulcer. Gastric volvulus was the most common finding, as it was detected in 12 patients (60%) (Fig. 6). All the previous abnormalities were managed as discussed in the “methodology” section during the same laparoscopic session.

Operative time had a mean value of 142 min, whereas intraoperative blood loss ranged between 50 and 200 ml. No operative complications were encountered in our study.

All patients had a regular (reviewer #1) postoperative course with a satisfactory improvement of gastrointestinal and respiratory manifestations, except one patient who developed postoperative hydro-pneumothorax. Chest tube drainage was required for 5 days. No postoperative mortality was encountered in our study. The duration of hospitalization ranged between 2 and 12 days (median = 3 days).

During follow-up, all patients reported significant improvement in their reported symptoms. At a median follow-up of 48 months (range 30–60 months), three patients (reviewer #1) developed radiological recurrence with no significant clinical symptoms.

As shown in Table 4, GIQLI, along with its individual parameters, showed a significant improvement after the operation compared to the corresponding preoperative scores.

**Table 4:** GIQLI changes before and after surgery

Test	Preoperative *n* = (20)			Postoperative *n* = (20)			*p* value	Change
	Mean	SD	Range	Mean	SD	Range		
GIQLI	76.5	8.2	61:91	100	7.3	86:112	< 0.001	Improved
Symptoms	40.1	5.5	31:48	56.2	7.4	41:64	< 0.001	Improved
Physical	12.2	3.2	8:18	17.2	1.4	4:19	< 0.001	Improved
Social	10.3	1.4	7:12	11.7	0.9	10:13	< 0.001	Improved
Emotional	10.2	1.8	7:13	11.9	1.1	9:13	< 0.001	Improved
Medical	2.95	0.8	1:4	3.4	0.7	2:4	0.001	Improved
Satisfaction	–	–	–	3.4	0.75	2:4	–	–

Although GIQLI showed a significant increase with subsequent follow-up visits (*p* < 0.001), its individual parameters showed no significant changes, apart from patient satisfaction, which showed similar changes to the total score (Table 5).

**Table 5:** Postoperative GIQLI stratified by time

Scoring test	0–1 years *n* = (18)			1–3 years *n* = (16)			Four years *n* = (14)			*p* value
	Mean	SD	Range	Mean	SD	Range	Mean	SD	Range	
GIQLI	105	6.2	93:114	105	4.8	98:113	108.8	4.6	100:117	< 0.001
Symptoms	58	6.4	44:66	59	5.2	46:66	60.2	4.5	52:66	0.472
Physical	18.3	1.4	16:20	17.9	1.3	16:20	19.1	1.5	16:21	0.067
Social	12.5	1.2	11:15	12.4	1.3	11:15	12.6	1.3	11:15	0.840
Emotional	13.1	4	12:16	12.9	5	12:16	13.4	1.5	12:18	0.640
Medical	3.4	0.7	2:4	3.5	0.6	2:4	3.6	0.5	3:4	0.525
Satisfaction	3.6	0.7	3:5	3.8	0.6	3:5	4.3	0.6	3:5	0.013

## Discussion

Diaphragmatic eventration represents a rare pathology with an incidence rate of 0.05% [14]. Most patients have their left hemidiaphragm affected rather than the right side, as reported in previous studies [4, 14], and that coincides with our findings which showed that 95% of the included participants had left-sided eventration.

Diaphragmatic eventration with late-onset symptoms in adulthood is exceedingly rare, with few cases reported in the literature. Most of the published articles in the existing literature are single case reports and small case series [9, 10].

Patients with diaphragmatic eventration typically present with respiratory manifestations, including orthopnea and exertional dyspnea, secondary to diaphragmatic elevation and ventilation/perfusion mismatch [15]. However, they may have gastrointestinal symptoms such as dyspepsia, reflux, dysphagia, and epigastric pain [14].

The current study is one of the largest series that handled the laparoscopic management of diaphragmatic eventration patients who complained mainly of gastrointestinal symptoms. That is an advantageous point in favor of our study as few reports of laparoscopic approach for the management of diaphragmatic eventration in adult patients have been published [5, 16].

Since the great majority of DE in adults are asymptomatic, these symptoms could be attributed to the accompanying gastrointestinal problems instead of to DE (reviewer #2).

The open surgical approach (thoracotomy or laparotomy) for diaphragmatic eventration has traditionally been preferred [17–19]. However, minimally invasive approaches, either thoracoscopy or laparoscopy, have proved their effectiveness in such cases as they have multiple advantages over the open approach [7, 20]. Whether to perform the plication procedure with thoracoscopy or laparoscopy depends mainly on the surgeon’s preference [7].

In patients with gastrointestinal manifestations, we think that laparoscopy has many advantages over thoracoscopy, mainly in the diagnosis and management of any concomitant intraabdominal pathology requiring surgical intervention at the time of eventration repair.

Regarding the diagnosis, we have an interesting finding to report, which is not mentioned in our results. In our center, by the routine CT evaluation for all patients (reviewer #1), one patient was diagnosed with a diaphragmatic hernia before the operation but turned out to have diaphragmatic eventration after laparoscopic assessment. In addition, two patients diagnosed preoperatively with diaphragmatic eventration turned out to be only diaphragmatic hernia that was managed by laparoscopy. The previous findings highlight the importance of laparoscopy in the confirmation of the diagnosis, especially in the presence of any radiological debate before the operation.

When it comes to the role of laparoscopy in management, we detected multiple anomalies that were successfully managed by laparoscopy during the same session. The most common finding beside eventration was gastric volvulus (60%).

As most eventration cases are left sided, this creates a wide left subphrenic space allowing rotation of the stomach and resulting in volvulus [21, 22]. It was previously reported that the previous combination is uncommon [6, 21]. Others considered symptomatic gastric volvulus in association with eventration an emergency surgical condition requiring repair [10, 23], as it is associated with high morbidity and mortality rates [24] secondary to gastric ischemia, necrosis and perforation [21, 24].

In our series, ten out of the included volvulus cases had chronic volvulus, while the remaining two had the acute type. All these patients were successfully managed with posterior gastropexy with significant improvement of their symptoms with no significant postoperative morbidity.

Interestingly, in our series, we also showed the effectiveness of the laparoscopic approach in the management of a dual diaphragmatic problem in five adult patients (three hiatus hernias and two diaphragmatic hernias). The association between eventration and hiatal hernia is rarely reported in the literature, and it was previously reported in a 2-month-old boy who was managed by plication and crural repair [25].

Overall, we see that laparoscopy is of great value in patients with diaphragmatic eventration and gastrointestinal problems, as it helps to manage both diaphragmatic and other intraabdominal problems.

It is recommended to perform larger studies with more eventration cases to elucidate the role of laparoscopy in such cases. In addition, these studies could compare laparoscopy with thoracoscopy to highlight the upper hand of laparoscopy in the management of this condition, especially if associated with gastrointestinal problems.

## Conclusion

The laparoscopic approach is an attractive, effective option for the surgical management of adult diaphragmatic eventration (reviewer #1). The surgeon should not only choose his approach based on his skills and expertise but also patient presentation should be considered.
